# Are pertussis cases reported too late for public health interventions? Retrospective analysis of cases in London and South East England, 2010 to 2015

**DOI:** 10.2807/1560-7917.ES.2017.22.29.30577

**Published:** 2017-07-20

**Authors:** Helen Crabbe, María Saavedra-Campos, Neville Q Verlander, Anusha Leonard, Jill Morris, Amanda Wright, Sooria Balasegaram

**Affiliations:** 1 Field Epidemiology Training Programme, Public Health England, London, United Kingdom; 2 European Programme for Intervention Epidemiology Training, European Centre for Disease Prevention and Control, Stockholm, Sweden; 3 Public Health England South East Centre, Chilton, United Kingdom; 4 Field Epidemiology Service, National Infections Service, Public Health England, London, United Kingdom; 5 Statistics, Modelling and Economics Department, National Infection Service, Public Health England, London, United Kingdom

**Keywords:** Pertussis, epidemiology, post-exposure prophylaxis, public health policy, surveillance, vaccine-preventable diseases

## Abstract

In the United Kingdom, pertussis guidance recommends prophylaxis for household contacts within 21 days of case symptom onset if the household includes a vulnerable contact. The aim of our study was to identify characteristics associated with cases reported late for public health action. We reviewed the epidemiology of cases reported in London and South East England for the period 2010 to 2015. We characterised risk factors associated with late reporting of cases and described public health actions taken on timely reported cases. From 2010 to 2015, 9,163 cases of pertussis were reported to health protection teams. Only 11% of cases were reported within 21 days of onset, limiting opportunities for secondary prevention. Timely reporting was associated with younger age groups, pregnancy, being a healthcare worker and being reported by schools or hospital clinicians. Late reporting was associated with older age groups and general practitioner or laboratory reporting. Delays, such as those due to insidious onset and late presentation to healthcare, may be unavoidable; however, delay in reporting once a patient presents can be reduced since cases can be reported before laboratory confirmation. Thus we recommend working with clinicians and laboratories to determine causes and improve early reporting to public health.

## Introduction

Pertussis, or whooping cough, is a highly infectious respiratory disease caused by the bacterium *Bordetella pertussis*. If left untreated, it is transmissible for up to 21 days from onset of cough. However, it becomes non-infectious after 5 days of antibiotic treatment [1]. Despite high levels of immunisation and vaccination coverage of over 95% in the United Kingdom (UK) [2], pertussis has recently re-emerged as a major public health threat [3,4] with a national outbreak in 2012 resulting in several infant deaths [5].

While pertussis was once considered a disease primarily affecting infants and children, many countries currently observe high rates of pertussis among older children, adolescents and adults [6-11]. The risk of severe illness and death is highest among infants younger than 1 year [5-7,11]. Serious illness is less common in older children and adults; however, they can transmit the infection to vulnerable contacts, including the unimmunised or incompletely immunised babies [1,3,7,12].

Timely diagnosis and management of cases are important to minimise transmission and severe disease. This relies on cases seeking medical attention early after the onset of cough and clinicians reporting on clinical suspicion. In England, contact tracing by health protection teams (HPTs) is conducted for cases with onset of cough less than 21 days before reporting (or, if they are a healthcare worker, any duration of cough). Household contacts are offered prophylaxis if any member of the household is in a priority group. Priority groups include vulnerable individuals at risk of severe complications, i.e. unimmunised infants younger than 1 year, individuals at increased risk of transmitting infection to vulnerable individuals, pregnant women (> 32 weeks in gestation), healthcare workers (HCWs) working with infants and pregnant women, people who work with infants too young to be fully vaccinated, and people who share a household with an infant too young to be fully vaccinated [5]. Anecdotal reports suggest that many such cases present late and are reported too late for public health action.

On presentation of a suspected case of pertussis, clinicians in general practice, school nurses, hospitals etc should verbally notify the responsible officer of the local HPT within 24 hours, and in writing within 3 days [5]. HPTs contact the clinician or patient by telephone to gather additional details. Dates of onset are given by the reporting person at the time of reporting or estimated if the actual date of onset is unknown. Positive samples from local laboratories are also notified to HPTs through daily electronic laboratory reporting. For these samples, onset date is derived from the laboratory record of received samples if it has been completed, and if not, is estimated by the case management tool as described below. For cases reported within 21 days of symptom onset, public health teams contact the case or healthcare provider to confirm the date of onset and determine if there are any vulnerable or priority contacts in the household who need prophylaxis.

The aim of our study was to quantify late reporting and identify characteristics associated with cases reported late for public health action in London and South East England using surveillance data for the period from 2010 to 2015.

## Methods

### Study design

We conducted a retrospective analysis in which we included all reported confirmed or probable cases of pertussis between 2010 and 2015 who were resident in the area served by our regional unit, i.e. London or South East England (defined as Public Health England region, which includes Thames Valley, Hampshire and the Isle of Wight, Surrey, Sussex and Kent).

### Definitions

According to national guidance [5], a confirmed case of pertussis is defined as any person with signs and symptoms consistent with pertussis and for whom *B. pertussis* has been isolated from a respiratory sample (typically a nasopharyngeal aspirate or nasopharyngeal/perinasal swab) or who have an anti-pertussis toxin IgG titre > 70 IU/mL (in the absence of vaccination within the past year) or for whom *B. pertussis* has been confirmed by PCR in a respiratory clinical specimen. Serology is only recommended for patients with a cough of at least 14 days [5].

A probable case is defined as any person in whom a clinician suspects pertussis or any person with an acute cough lasting for 14 days or more without an apparent cause, plus one or more of the following: paroxysms of coughing or post-tussive vomiting or inspiratory whoop, in the absence of laboratory confirmation or epidemiologic link to a laboratory-confirmed case.

We defined a late case of pertussis as a probable or confirmed case reported to the HPTs more than 21 days from the onset of cough, either due to late presentation to healthcare or late reporting by clinicians. Timely cases were defined as those reported within 21 days of onset of cough.

### Data extraction

We extracted data on confirmed and probable cases reported on the HPT’s case management system (HPZone, by Infact, Shipley) including data on demographics, vaccination status, date of report (‘date entered’), date of onset, source of reporting, and occupation of the patient. We imported the data to MS Excel and STATA v12. Duplicates were identified by checking names, date of birth (DOB) and National Health Service (NHS) numbers, and the record with the larger proportion of completed fields was retained.

When entering the onset date onto HPZone, the user is required to select their confidence in the onset date. The chosen confidence level determines the onset date recorded by the system. To remove uncertainties around the estimated date of onset, we retained only those cases with either an observed onset date or dates recorded with fair and high confidence.

Data on public health management could only be obtained by manually reviewing individual case notes. Owing to the large number of case notes, we were only able to review the timely cases reported in 2015, including validation of the date of onset. Cases without further information on the onset date were excluded as the date could not be validated. 

Administrative boundaries were obtained from corporate shapefiles kept on the central geographical information system (GIS) server, using Esri’s ArcView v10.2. The index of multiple deprivation (IMD) 2015 score [13] was obtained for each case through postcode matching to lower super output area (LSOA). IMD is the official measure of relative deprivation for small areas or neighbourhoods (LSOAs, with 1,000–3,000 residents) in England and ranks every small area from 1 (most deprived) to 32,844 (least deprived). It combines information from seven domain indices (income, employment, education, health, crime, access to housing and services, and living environment) to produce an overall relative measure of deprivation, with a quintile score from the first quintile representing the least deprived (group 1) to the fifth quintile representing the most deprived (group 5) areas [14]. Population demographics were based on the Office of National Statistics (ONS) 2014 mid-year estimates for HPT areas [15].

### Descriptive epidemiology

We characterised demographics (age, sex and geographical location by HPT area), confidence of diagnosis (confirmed or probable), deprivation score, pregnancy status, source of report and occupation of the patient (HCW or education worker) between 2010 and 2015. Incidence rates were calculated for the < 1, 1–9, 10–19, 20–39, 40–59 and ≥ 60 year-old age groups using the residential population as denominator. We calculated mean incidence by dividing the number of cases occurring per year by the resident population per HPT area [15] for the study period and plotted it on a map.

The proportions of unknown, timely and late classifications described in the validated dataset for 2015 were applied to the previous years and presented graphically to plot the estimated number and proportion of timely and late cases across the whole study period.

For timely reported cases in 2015, we examined case notes for the public health action taken and types of vulnerable and priority contacts identified.

### Statistical analysis

We conducted an analysis of the characteristics potentially associated with being classified as a late case in the validated dataset of confirmed and probable pertussis cases reported in 2015. We performed single variable analysis using Pearson’s chi-square test, or Fisher’s exact test. All variables with a significance value of p < 0.2 were included in a logistic regression model along with a priori confounders of age, sex, IMD, season and source of report but omitting those for which more than 20% of individuals had no information. We used a backwards stepwise approach to identify a final model, eliminating variables with the highest p value first from a likelihood ratio test and identifying possible confounders. If a variable did not improve the model it was removed, therefore adjusted odds ratio (aOR) are not available for these predictors. IMD aOR were calculated to show difference of lateness by IMD categories after adjustment by other confounders. Variables with more than 20% missing data were added to the model at the end of the model-building process to check if they improved the model, as their earlier inclusion would have had a negative impact on the model-building process because fewer observations were available in the complete case analysis. 

### Sensitivity analysis

To check if the effect estimates remained for other years, the statistical analysis was re-run on the unvalidated data from 2010 to 2014, using the initially recorded onset date, again retaining only the cases with observed date and date recorded with fair and high confidence.

## Results

We identified 9,311 confirmed and probable cases of pertussis reported from 2010 to 2015. Of these, 10 cases had been denotified and 138 were duplicates, leaving a total of 9,163 cases of whom 7,088 (77%) were confirmed and 2,075 (23%) were probable (Table 1).

**Table 1:** Demographics and characteristics of pertussis cases reported to health protection teams in London and South East England, 2010–2015 (n = 9,163)

Variable	Number		%	Confirmed	% (row)	Probable	% (row)
Total	9,163		100	7,088	77	2,075	23
Sex							
Male	4,113		45	3,224	78	889	22
Female	5,041		55	3,859	77	1,182	23
Information missing	9		1	5	NA	4	NA
Age group							
Newborn (< 1 month)	101		1	72	71	29	29
1–3 months	386		4	251	65	140	36
4–11 months	146		2	40	27	106	73
1–9 years	894		10	358	40	536	60
≥ 10 years	7,616		83	6,357	83	1,259	17
Information missing	20		1	10	NA	5	NA
Year of notification							
2010	140		2	85	61	55	39
2011	359		4	267	74	92	26
2012	3,688		40	2,644	72	1,044	28
2013	1,776		19	1,461	82	315	18
2014	1,551		17	1,275	82	276	18
2015	1,649		18	1,356	82	293	18
Occurred in outbreak year (reported in 2012)?							
No		5,475	40	4,444	81	1,031	19
Yes	3,688		60	2,644	72	1,044	28
Season							
Spring	1,700		19	1,311	77	389	23
Summer	2,327		25	1,901	82	426	18
Autumn	3,171		35	2,418	76	753	24
Winter	1,965		21	1,458	74	507	26
Geographical location (health protection team name)							
Kent	740		8	708	96	32	4
Outside London and South East England	18		1	16	89	2	11
Thames Valley	1,266		14	700	55	566	45
Wessex	1,282		14	1,039	81	243	19
Sussex/Surrey	2,277		25	2,043	90	234	10
North-east and central London	1,073		12	762	71	311	29
North-west London	616		7	436	71	180	29
South-east London	594		6	387	65	207	35
South-west London	1,256		14	959	76	297	24
Information missing	41^a^		1	38	NA	3	NA
Location/status							
At home	8,440		92	6,428	76	2,024	24
In hospital	100		1	57	57	43	43
Deceased	6		1	6	100	0	0
At a temporary address	10		1	5	50	5	50
Information missing	607		7	592	NA	3	NA
Hospitalised?							
No	968		11	450	47	518	54
Yes	280		3	184	66	96	34
Information missing	7,915		86	6,454	NA	1,461	NA
Ethnicity							
White	296		3	144	49	152	51
Non-white	112		1	43	39	69	62
Information missing	8,755		96	6,901	NA	1,854	NA
Case status							
Probable	2,075		23	NA	NA	NA	NA
Confirmed	7,088		77	NA	NA	NA	NA
Deprivation (index of multiple deprivation 2015 quintiles)							
1 (least deprived)	808		9	559	69	249	31
2	1,510		16	1,114	74	396	26
3	1,733		19	1,357	78	376	22
4	2,086		23	1,605	77	481	23
5 (most deprived)	2,967		32	2,405	81	562	19
Information missing	59		1	48	NA	11	NA
Recent travel to another country?							
Not travelled	599		7	289	48	310	52
Travelled	169		2	88	52	81	48
Information missing	8,395		92	6,711	NA	1,684	NA
Sex/pregnancy status							
Male	4,113		45	3,224	78	889	22
Female, not pregnant	5,022		55	3,851	77	1,171	23
Female, pregnant	29		1	13	45	16	55
Vaccinated							
No	147		2	69	47	78	53
Yes	449		5	206	46	258	58
Information missing	8,567		93	6,813	NA	1,739	NA
Occupation							
Works in healthcare?							
No	9,048		99	7,030	78	2,018	22
Yes	115		1	58	50	57	50
Works in education?							
No	9,132		100	7,075	77	2,057	23
Yes	31		1	13	42	18	58
Source of notification							
Health protection team	39		1	30	77	9	23
General practitioner	2,136		23	655	31	1,481	69
Hospital	523		6	294	56	229	44
Laboratory report	5,775		63	5,720	99	55	1
School	38		1	17	45	21	55
Other	652		7	372	57	280	43

NA: not available, not applicable or not known.

^a^ Location information was missing if the case subsequently moved out of the study area.

### Epidemiology of cases in London and South East England in 2010–2015

**Figure 1:**
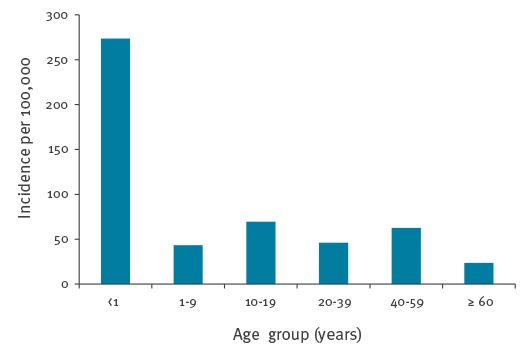
Incidence rate, per 100,000 population, for reported pertussis cases, by age group, London and South East England, 2010–2015 (n = 9,163)

The majority of cases were older than 10 years (83%) (Table 1). The incidence was highest in those younger than 1 year (273 cases/100,000 population) (Figure 1).

**Figure 2:**
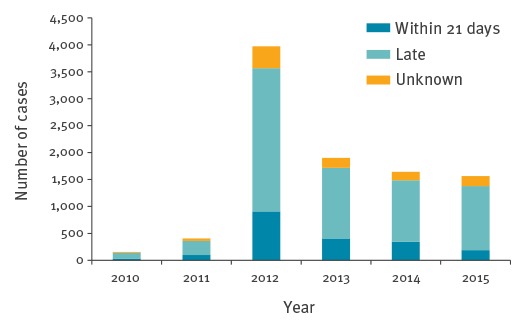
Cases of pertussis reported to health protection teams and proportion reported late, per year, London and South East England, 2010–2015 (n = 9,163)

In 2012, the outbreak year, 40% (n = 3,688) of the total cases were reported. The number of cases remained steady in subsequent years, with around 1,600 cases reported each year (2013–15) (Figure 2). There is seasonal fluctuation of cases, with the highest number occurring in the autumn (35%). There were six deaths (including five in infants under the age of 1 year); none of the deceased cases were reported late.

Proportions reported late, timely and unknown are corrected for 2010–14 using proportions found in the validated 2015 data.

**Figure 3:**
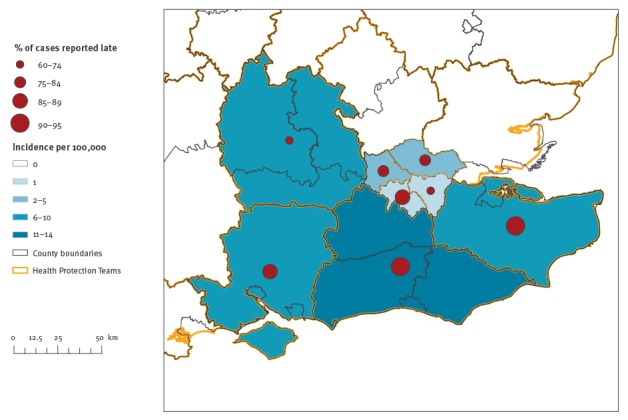
Mean incidence of pertussis cases per 100,000 per year reported to health protection teams (HPT) in 2010–2015 (n = 9,163), and proportion reported late in 2015 by HPT area (n = 1,649), London and South East England

The mean incidence of pertussis cases between 2010 and 2015 per HPT area ranged from 1 to 14 cases per year per 100,000 population (Figure 3). The proportion of cases increased with the deprivation index, with 32% in the most deprived group compared with 9% in the least deprived (Table 1). There was no difference in the expected proportions in deprivation quintiles for the cases younger than 1 year.

### Validation of the cases reported late, using 2015 data

In 2015, 1,649 cases were notified. From the recorded onset date, 619 (38%) were classified as timely, 973 (59%) were late and 57 cases had no onset date recorded (3%). Of the timely cases, detailed case notes were examined for 595; case notes for the others were not available as they were no longer residents of London or South East England, and thus their records would have been transferred to another region. In 187 cases (31%), there was no further information, most often because cases were notified on confirmation by serology (taken after cough of at least 14 days’ duration) and therefore considered to be reported after 21 days [5]. Thus, although most unvalidated cases were likely to be late, they were removed from analysis as we could not assume this was the situation for all of them. Our final dataset for 2015 included 1,376 (83%) cases. Of these, 189 (14%) were reported in a timely manner (within 21 days) and 1,187 (86%) were reported late (Table 2).

**Table 2:** Number of pertussis cases reported and comparison of demographics and characteristics for non-late and late reporting groups, London and South East England, 2015 (n = 1,649)

**Variable**	**Non-late**		**Late**		**Total**	**Missing**	** Unadjusted OR ** **(95% CI)**	**p value** **(chi-square or Fisher’s exact test)**	** AOR (95% CI) ** **(adjusted for age,** **source of report and** **laboratory confirmation)**	**p value** **(likelihood ratio test)**
	**n**	**% (column)**	**n**	**% (column)**	**n**	**n**				
Number	189	11 (row)	1,187	72 (row)	1,649^a^	273	NA	NA	NA	NA
Validated cases (without missing data)	189	14 (row)	1,187	86 (row)	1,376	0	NA	NA	NA	NA
Sex										
Male	84	44	521	44	605	125	Ref	0.887	NA	NA
Female	105	56	666	56	771	148	1.02 (0.75–1.39)		NA	NA
Age group										
Newborn (< 1 month)	4	2	1	0.1	5	0	Ref	**< 0.001**	Ref	**< 0.001**
1–3 months	27	14	15	1	42	5	2.22 (0.23–21.7)		1.19 (0.11–12.4)	
4–11 months	17	9	6	0.5	23	0	1.41 (0.13–15.3)		1.48 (0.13–17.2)	
1–9 years	45	24	112	9	157	19	**9.96** **(1.08–91.5)**		7.80 (0.79–79.1)	
≥ 10 years	95	50	1,052	89	1,147	249	**44.3** **(4.90–400)**		**14.4** **(1.48–139)**	
Information missing	1	0	1	0	2	0	NA		NA	
Season										
Spring	38	20	273	23	311	49	Ref	0.277	NA	NA
Summer	57	30	299	25	356	82	0.73 (0.47–1.14)			
Autumn	46	24	344	29	390	97	1.04 (0.66–1.65)			
Winter	48	25	271	23	319	45	0.79 (0.50–1.24)			
Geographical location (health protection team name)										
Kent	10	5	121	10	131	16	Ref	**0.032**	NA	NA
Thames Valley	13	7	44	4	57	48	**0.28** **(0.11–0.68)**			
Wessex	21	11	143	12	164	55	0.56 (0.26–1.24)			
Sussex / Surrey	26	14	304	26	330	95	0.97 (0.45–2.06)			
North-east and central London	28	15	121	10	149	1	**0.36** **(0.17–0.77)**			
North-west London	23	12	110	9	133	1	**0.40** **(0.18–0.87)**			
South-east London	29	15	93	8	122	1	**0.27** **(0.12–0.57)**			
South-west London	39	21	244	21	283	51	**0.52** **(0.25–1.07)**			
Information missing	0	0	5	0	5	5	NA			
Hospitalised?										
No	47	70	88	91	135	4	Ref	**0.001**	NA	NA
Yes	20	30	9	9	29	1	**0.24** **(0.09–0.61)**			
Information missing	122	NA	1,090	NA	1,212	268	NA			
Case status										
Probable	118	62	168	14	286	7	Ref	**< 0.001**	**Ref**	**< 0.001**
Confirmed	71	38	1,019	86	1,090	266	**10.1** **(7.20–14.1)**		**2.96** **(1.92–4.58)**	
Deprivation (index of multiple deprivation 2015 quintiles)										
1 (least deprived)	25	13	110	9	135	14	Ref	**0.002**	Ref	0.943
2	51	27	210	18	261	29	0.94 (0.55–1.59)		0.94 (0.47–1.86)	
3	41	22	231	20	272	44	1.28 (0.74–2.21)		0.87 (0.44–1.74)	
4	29	15	247	21	276	55	**1.94** **(1.08–3.46)**		1.11 (0.54–2.28)	
5 (most deprived)	43	23	382	32	425	128	**2.02** **(1.18–3.45)**		0.90 (0.46–1.78)	
Information missing	0	NA	7	NA	7	3	NA		NA	
Recent travel to another country?										
Not travelled	30	79	46	72	76	3	Ref	0.428	NA	NA
Travelled	8	21	18	28	26	0	1.47 (0.52–4.40)			
Information missing	151	NA	1,123	NA	1,274	270	NA			
Sex/pregnancy status										
Male	84	44	521	44	605	125	Ref	0.887	NA	NA
Female, not pregnant	101	53	661	56	762	148	1.06 (0.77–1.44)			
Female, pregnant	4	2	5	0.5	9	0	**0.20** **(0.05–0.77)**			
Occupation										
Works in healthcare?										
No	185	98	1,179	99	1,364	271	Ref	**0.048**	NA	NA
Yes	4	2	8	1	12	2	**0.31** **(0.08–1.44)**			
Works in education?										
No	188	100	1,184	99.7	1,372	273	Ref	0.512	NA	NA
Yes	1	0.5	3	0.3	4	0	0.48 (0.04–25.1)			
Source of notification										
Hospital	41	22	39	3	80	3	Ref	**< 0.001**	Ref	**< 0.001**
General practitioner	99	52	212	18	311	16	**2.25** **(1.37–3.70)**		1.18 (0.61–2.27)	
Laboratory report	10	5	852	72	862	242	**89.6** **(41.8–191)**		**20.5** **(8.52–49.6)**	
School	5	3	1	0.1	6	1	0.21 (0.02–1.88)		**0.54** **(0.01–0.52)**	
Other	34	18	82	7	116	11	**2.54** **(1.40–4.59)**		1.23 (0.60–2.53)	

AOR: adjusted odds ratio; CI: confidence interval; NA: not available, not applicable or not known; Ref: reference group for comparison.

Numbers in bold indicate a significant result at the p < 0.05 level.

Analysis does not include missing data.

^a ^This total includes cases with missing information on timeliness.

### Single variable analysis

Cases older than 1 year had higher odds of being reported late. Being a confirmed case (odds ratio (OR) = 10.1; 95% confidence interval (CI): 7.20–14.1, compared with probable) and higher deprivation quintiles (e.g. quintile 5; OR = 2.02; 95% CI: 1.18–3.45, compared with quintile 1) were associated with late reporting. Cases reported by a hospital clinician were more likely to be timely, compared with a general practitioner (GP) or a laboratory report as the source (Table 2). Being hospitalised at the time of reporting, working in healthcare (OR = 0.31; 95% CI: 0.08–1.44) or education, or being pregnant was also associated with timely reporting (Table 2).

### Multivariable analysis

In the final adjusted multivariable model, adjusted for age, laboratory confirmation and source of report, being 10 years or older (aOR = 14.4; 95% CI: 1.48–139), being a confirmed case (aOR = 2.96; 95% CI: 1.92–4.58), and source being a laboratory report (aOR = 20.5; 95% CI: 8.52–49.6) compared with reporting from hospital clinician, were all significantly associated with being reported late. Conversely, cases reported by schools were more likely to be timely (Table 2).

In the sensitivity analysis using data from 2010 to 2014, similar effects were found. Age (e.g. ≥ 10 years: OR = 7.31; 95% CI: 4.18–12.8, compared with newborns (< 1 month-old)), being a confirmed case (aOR = 2.03; 95% CI: 1.72–2.42), and source being a laboratory report (aOR = 2.19; 95% CI: 1.68–2.85) compared with reporting from hospital clinicians, all remained significant.

Using the larger dataset we also found that being reported in autumn as opposed to spring (aOR = 1.25; 95% CI: 1.08–1.46) was associated with late reporting and being a HCW with early reporting (aOR = 0.54; 95% CI: 0.35–0.83), also adjusted for HPT area. The number of unknown, timely and late cases in other years (2010–14) was estimated in Figure 2, using the validated corrections to the original proportions of lateness found.

### Public health action taken on timely reported cases, 2015 dataset

For the 619 cases reported within 21 days of symptom onset in 2015, a risk assessment was performed which found that 31% (n = 189) required contact tracing. Vulnerable and/or priority contacts were identified for 20 of those cases (11%), of whom 18 were recorded as having contacts that were advised to take prophylaxis. The types of high-risk and/or vulnerable contacts encountered were mostly HCW and infants younger than 1 year (Table 3).

**Table 3:** Number of vulnerable and priority contacts identified for timely confirmed and probable cases of pertussis, London and South East England, 2015 (n = 595^a^)

**Type of priority contact**	**n**	**%**
Contacts at risk of transmitting to vulnerable contact		
Healthcare workers	11	41
Pregnant women > 32 weeks	2	7
Prolonged contact with infants < 4 months	1	4
Vulnerable contacts, increased risk of severe disease		
Infants < 1 year in household	7	26
Other/unspecified vulnerable contact in household	4	15
Infant < 4 months in household	2	7
**Subtotal^ b^**	**27**	**100**
No priority contacts identified	568	95
**Total^ b^**	**595**	**100**

^a^ 595 of the 619 cases had detailed records for review.

^b^ Some cases had more than one priority or vulnerable contact.

## Discussion

The reported incidence of pertussis was highest in the age group of under 1 year-olds which is consistent with previous findings [3,5,6,16-18]. However, as disease is more likely to be severe in those aged under 1 year, often requiring hospitalisation, our study found that this group was more likely to be reported in a timely manner.

Some changes in incidence over time are likely to be due to changes in laboratory testing methods. The rise in cases before the outbreak in 2012 was due to improved ascertainment in older age groups and the introduction of serology testing in 2001 [5,19]. The sustained number of cases after 2012 is likely to be due to better awareness of reporting, but also improved diagnostics and laboratory testing (PCR, serology and oral fluid). Since 2014, regional laboratories have offered a pertussis PCR service for patients in all age groups in both hospital and primary care settings [5]. A national oral fluid testing service was also introduced in January 2013 [5].

In our study, we identified that 86% of cases in 2015 were reported to the HPT more than 21 days after date of onset, with 63% of all cases reported by laboratories. Serology testing can only be used two weeks after symptom onset, so by the time the result is reported, it is likely to be received after the 21-day window, hence HPTs risk assess all these cases as late. The serological assay is targeted towards older children and adults [5], which could explain the higher risk of lateness in older age groups.

Increased odds of late reporting for confirmed cases compared with probable cases suggest that clinicians may not always be reporting on clinical suspicion but on laboratory confirmation. There are also higher odds of late reporting from laboratory reports and by GPs compared with hospital clinicians (aOR = 20.5; 95% CI: 8.52–49.6; p < 0.001). There may be practical considerations in waiting for laboratory confirmation. A clinician may not initially report cases with mild symptoms and insidious onset if pertussis is only a differential diagnosis. A physician who suspects pertussis as a minor differential diagnosis may not report every time they order a pertussis test. However, according to public health regulations [20], when clinicians suspect pertussis as a probable or the most likely diagnosis, they should report on clinical suspicion regardless of the accuracy of their diagnosis; thus reporting in this subset could be improved. In addition, clinicians can also report cases as ‘possible’ where pertussis is not thought to be the most likely diagnosis. In the UK, immediate public health actions are only completed for confirmed and probable cases. Reporting of possible cases would allow for timely follow-up of laboratory results, and therefore, actions may be taken if a possible case later becomes a confirmed case.

Our study shows that there are more reported pertussis cases in the more deprived areas, but this did not occur in cases younger than 1 year. Late reporting was not related to deprivation after adjustment. The increased incidence in more deprived groups may reflect service use, access, vaccine uptake, living conditions or other determinants of health.

Younger age groups, HCWs, education workers and pregnant women were more likely to be reported in time, suggesting that clinicians do recognise the importance of public health interventions to prevent severe disease in vulnerable groups.

Prophylaxis of contacts was indicated for 11% of cases reported in a timely fashion in 2015. Although effectiveness of secondary prophylaxis is limited [21], it is still important to administer in order to prevent severe disease in vulnerable contacts or transmission to the vulnerable from priority contacts. English guidance limits prophylaxis to those who need it [5] but, compared with vaccination, it is a measure which controls disease by preventing secondary transmission.

Chen and Orenstein [22] suggest that owing to a number of biases, cases of disease reported to surveillance systems are not random and reported cases are more likely to be more severe. It is not known how representative the cases included in this study are, although findings are in line with other studies [6,7,11]. Despite legal requirements on clinicians to notify pertussis cases on clinical suspicion [20], HPTs would not necessarily be notified of all community cases of pertussis, so the true incidence and prevalence of pertussis is unknown. However, similar epidemiological findings were seen in Barcelona in the period from 2009 to 2012 [6], where 82% of cases were laboratory-confirmed: Similar incidence rates (1.2–6.3/100,000 person-years) were reported, and most confirmed cases were under 1 year-old (87.9%). Hospitals reported the majority of cases (72%), reporting more confirmed cases than suspected cases (aOR = 2.8; 95% CI: 1.7–4.6; p < 0.05, compared with primary care centres). We found a similar proportion of 77% confirmed and 23% probable cases.

Pertussis is part of the infant vaccination programme in England. A pertussis-containing vaccine (5-in-1 vaccine, DTaP/IPV/Hib) is offered to infants at 2, 3 and 4 months of age [18]. A booster dose of pertussis-containing vaccine is given to children from 3 to 5 years of age. We have shown that a third (34%) of the cases younger than 4 months were reported late (Table 2), at an age with a greater risk of severe disease, as they would not yet have received the full course of vaccinations. This higher risk continues in the partially vaccinated group of 4–11-month-olds, with 26% of cases being reported late. 

Maternal vaccination is key to reducing disease in neonates. In 2016, Public Health England recommended a change of schedule for the maternal vaccination programme (in place since 2012) so that the vaccine is now offered between 16 and 32 weeks of gestation [5,18], as evidence supported effectiveness [23,24] even if given earlier in pregnancy than the third trimester, and it is hoped this will also improve uptake.

Most cases are reported too late for public health intervention. Late cases are likely to be an amalgamation of late presentation to clinical services and late diagnosis, or late reporting by the person assessing the case, e.g. cases where pertussis is a differential diagnosis, or awaiting laboratory confirmation when the diagnosis should be made on clinical grounds. This level of information is not routinely recorded, and so it is difficult to allocate cases accurately to these categories. Some cases will always present late, for instance because of the milder nature of the disease in adults and older children. Improvements should focus on the cases where it is possible to notify earlier, e.g. on high probability on clinical suspicion rather than waiting for laboratory confirmation.

### Limitations

Our study has a number of limitations. Firstly, the dataset may be incomplete and does not necessarily represent all pertussis cases, and under-reporting is likely to occur for milder cases. For practical reasons, we could only validate onset date for one year’s worth of data (2015). We chose the most recent whole year to be representative of current practice. Initial dates of onset may not be accurate. However, they are likely to be verified when the risk assessment takes place. Therefore, misclassification of cases as timely or late is likely to be small.

Using ‘date entered’ as a proxy for reporting date is likely to reflect accurately the reporting date and if not, the difference is unlikely to be by more than one day, having a small effect on our estimates. A case reported on serology is highly likely to be late, although we excluded these. Therefore, our calculated proportion of late cases is likely to be underestimated.

## Conclusions

Although it is encouraging that cases in young, hospitalised or pregnant individuals or in HCWs were reported in time for public health management, many cases were reported late either due to delays in presentation to health services or late reporting by the clinician. Exploring the reasons for late reporting could help understand the high levels of late reporting described in the study.

When implemented, public health interventions, including contact tracing, identified a small number of vulnerable and/or priority contacts in the 2015 cases. Although secondary prophylaxis is recognised as having a limited effect in preventing secondary transmission [5,21], given the potential severity of disease in vulnerable contacts, it is still considered essential to protect the very vulnerable by the use of prophylaxis. Thus the need to renew efforts for vaccination of the very young and vulnerable populations and to improve early reporting is apparent.

Education of GPs and clinicians on the importance of reporting cases in a timely manner and regular reminders to key audiences communicating the risk of late reporting of cases should occur. This includes feedback to GP groups to encourage reporting on clinical suspicion. Communicating the risk factors for late reporting and targeting health services providing care for the very young, unimmunised and vulnerable will help to address the differences in reporting. This should include GP clinical commissioning groups for local health services, HCWs and workers coming into contact with high-risk groups (nurseries, childcare, schools and maternal services).
